# Neuronal coordination of motile cilia in locomotion and feeding

**DOI:** 10.1098/rstb.2019.0165

**Published:** 2019-12-30

**Authors:** Milena Marinković, Jürgen Berger, Gáspár Jékely

**Affiliations:** 1 Living Systems Institute, University of Exeter, Stocker Road, Exeter EX4 4QD, UK; 2 Max Planck Institute for Developmental Biology, 72076 Tübingen, Germany

**Keywords:** ciliary band, ciliary swimming, marine larva, calcium, *Platynereis*, evolution

## Abstract

Efficient ciliary locomotion and transport require the coordination of motile cilia. Short-range coordination of ciliary beats can occur by biophysical mechanisms. Long-range coordination across large or disjointed ciliated fields often requires nervous system control and innervation of ciliated cells by ciliomotor neurons. The neuronal control of cilia is best understood in invertebrate ciliated microswimmers, but similar mechanisms may operate in the vertebrate body. Here, we review how the study of aquatic invertebrates contributed to our understanding of the neuronal control of cilia. We summarize the anatomy of ciliomotor systems and the physiological mechanisms that can alter ciliary activity. We also discuss the most well-characterized ciliomotor system, that of the larval annelid *Platynereis*. Here, pacemaker neurons drive the rhythmic activation of cholinergic and serotonergic ciliomotor neurons to induce ciliary arrests and beating. The *Platynereis* ciliomotor neurons form a distinct part of the larval nervous system. Similar ciliomotor systems likely operate in other ciliated larvae, such as mollusc veligers. We discuss the possible ancestry and conservation of ciliomotor circuits and highlight how comparative experimental approaches could contribute to a better understanding of the evolution and function of ciliary systems.

This article is part of the Theo Murphy meeting issue ‘Unity and diversity of cilia in locomotion and transport’.

## 1. Introduction

Ciliary locomotion occurs in the majority of unicellular eukaryotes [1,2] and is also widespread in animals. Animals can either swim or glide with cilia, both at larval stages and as adults. There is a great diversity in the mode of movement, the type of ciliation and the tissue-scale dynamics of cilia. Ciliary swimming is most common in the larval stages of marine invertebrates. The majority of bottom-dwelling marine invertebrate animals have a ciliated larval stage. These animals undergo a planktonic-to-benthic transition as part of their biphasic life cycle [3]. Ciliary gliding is often found in adult forms such as flatworms or placozoans where ciliary activity co-occurs with muscle-based or epithelial contractility [4,5]. Many animals also use cilia to generate feeding currents to capture food particles. Planktonic ciliary swimmers that also feed with cilia can display the most complex ciliary dynamics and have trade-offs between swimming and feeding [6].

In ciliary swimmers, gliders and feeders, the activity of cilia can change in response to environmental cues and is generally under nervous system control. For example, many ciliary swimmers can change their trajectory to move towards a light source by phototaxis [7]. Circadian or sensory-induced adjustments in ciliary beating allow planktonic organisms to regulate their depth in the water column [8]. There are several other contexts where ciliated fields and the flows they generate are important for animal physiology, including the establishment of symbiosis in squid [9], mixing the boundary layer in corals [10] or the movement of cerebrospinal fluid in the vertebrate brain [11]. All these activities require the coordination of multiple cilia across large ciliary fields, sometimes spanning the entire body. Here, we focus on the anatomical and functional organization of ciliary locomotor and feeding systems in invertebrates. We discuss different phenomena of ciliary coordination in ciliary bands and epithelia and the mechanisms of nervous system control. In some cases, large neurons known as ciliomotor neurons that innervate multiple ciliated cells are used to coordinate ciliary activity throughout an organism. The recently characterized whole-body ciliomotor circuit of the marine annelid *Platynereis dumerilii* [12] highlights the sophistication of a dedicated ciliomotor circuit. In *Platynereis* larvae, large biaxonal neurons form a morphologically and functionally distinct ciliomotor nervous system coordinating whole-body ciliary activity. We review the evidence suggesting that other ciliated larvae also have dedicated circuitry for the control of cilia. Future comparative studies could test the hypothesis that ciliomotor nervous systems have a unique evolutionary history with potentially deep origin in animal evolution [13,14].

## 2. Types of ciliary locomotor and feeding systems in invertebrates

**Figure 1.**
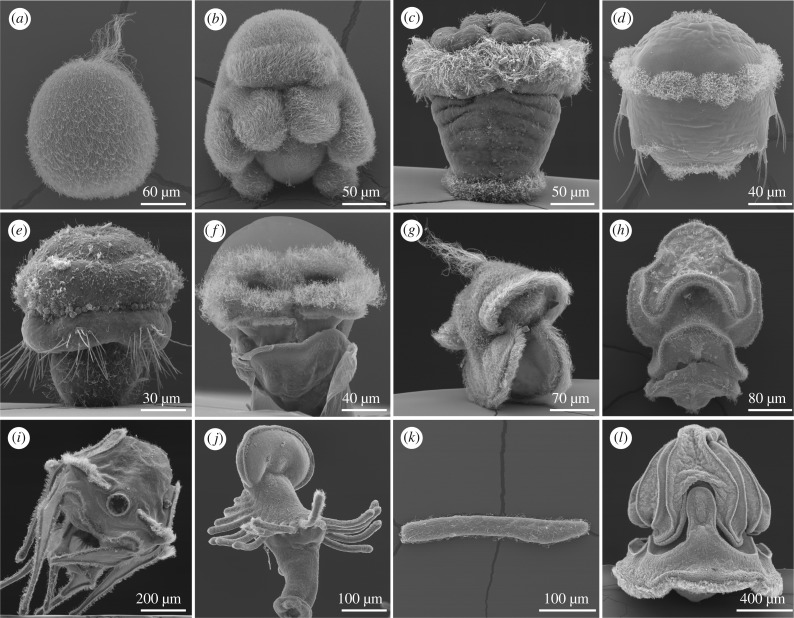
The diversity of ciliated larvae. (*a*) *Nematostella vectensis* uniformly ciliated planula (cnidarian), (*b*) Mueller's larva of the flatworm *Maritigrella crozieri,* uniformly ciliated, (*c*) annelid trochophore with ciliary bands, (*d*) annelid trochophore with ciliary bands (*P. dumerilii*), (*e*) larva of the brachiopod *Terebratalia transversa*, (*f*) *Aplysia californica,* mollusc veliger with ciliary bands, (*g*) *Lineus longissimus,* nemertean pilidium larva, (*h*) starfish bipinnaria larva, (*i*) echinoderm 8-arm-larva (sea urchin), (*j*) phoronid actinotroch larva, (*k*) amphioxus chordate larva and (*l*) *Schizocardium californicum* hemichordate tornaria.

**Figure 2.**
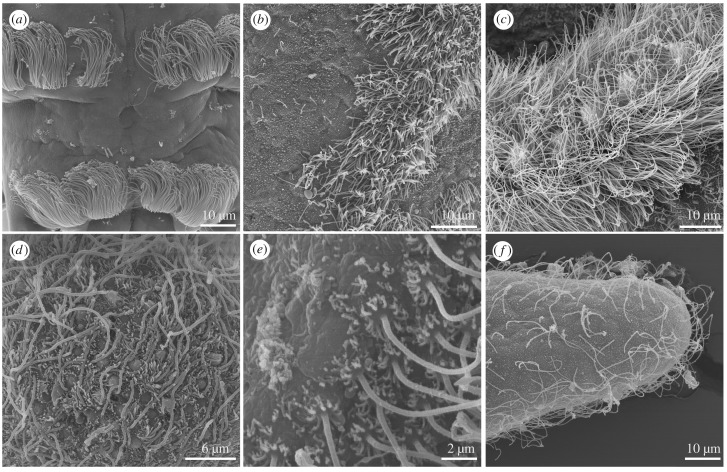
Mono- and multiciliated surfaces. (*a*) Annelid multiciliated cells of the ciliary band (*P. dumerilii*). (*b*) Multiciliated cells on a hemichordate larva. (*c*) Multiciliated cells on a nemertean pilidium larva. (*d*) Monociliated epithelium in the planula of *N. vectensis.* (*e*) Monociliated cells on echinoderm larval arms. (*f*) Monociliated cells in an amphioxus larva.

Ciliary systems occur either as uniformly ciliated body surfaces or as ciliary bands with more densely concentrated cilia that run around the body or along appendages (figures 1 and 2). Cilia in ciliary bands often emanate from specialized multiciliated cells, distinct from monociliated epithelial cells. Ciliary bands often have a dual role, enabling the animal to both swim and feed.

### (a) Locomotor cilia

Locomotor cilia occur in both larval and adult stages of invertebrates. Larval ciliary swimmers are present in many sponges and cnidarians, most spiralians, echinoderms, hemichordates and cephalochordates [15]. Ciliary swimming in adults is present in ctenophores, some flatworms and rotifers. Ciliary gliding is characteristic of placozoans and also occurs in some species of annelids, flatworms, nemerteans, gastrotrichs, gnathostomulids, gastropods and xenacoelomorphs [16–18].

**Figure 3.**
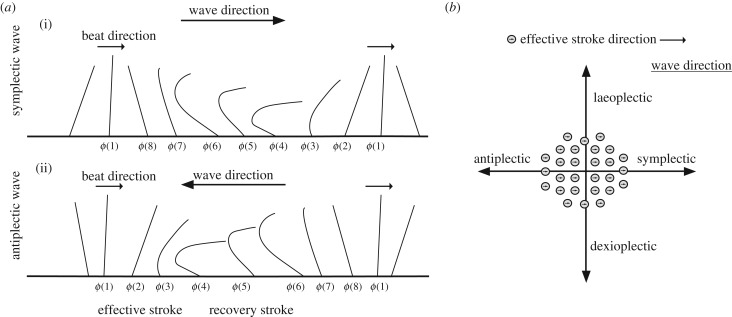
Types of metachrony. (*a*) Side view of a row of beating cilia. Symplectic metachronal waves (i) propagate in the same, while antiplectic waves (ii) propagate in the direction opposite to the direction of the effective stroke. (*b*) Top view of a bundle of cilia. Metachronal waves can propagate orthogonally to the beat plane. Laeoplectic waves propagate to the left, and dexioplectic to the right relative to the effective stroke of the cilia. Based on [19].

There is a great diversity in the patterns of ciliation and the mode of ciliary beating across animals (figure 1). Locomotor cilia can occur either on ciliated epithelia (e.g. placozoans, flatworms, sponge, cnidarian and cephalochordate larvae) or organized in discrete ciliary bands (most lophotrochozoan and echinoderm larvae, ctenophore combs). The ciliated cells can either have one (sponges, cnidarians, the annelid *Owenia,* echinoderms) or multiple cilia (most lophotrochozoan larvae, sponge trichimella larvae) (figure 3). Both types have a broad phyletic distribution and it is currently unclear if multiciliation evolved multiple times independently. The molecular pathways driving centriole amplification in multiciliated cells are well understood, and it was experimentally demonstrated that changes in the levels of expression of genes involved in centriole amplification can induce multiciliation [20]. It may be that the fine-tuning of these pathways led to the repeated emergence of multiciliation during evolution.

Cilia can be simple or compound, with compound cilia linked by filamentous bridges and able to support a larger body size and greater swimming speed [21]. Among animals, compound cilia occur in ctenophores, the largest ciliary swimmers [17]. The compound cilia in ctenophore comb plates are structurally complex, with multiple cilia grouped in bundles and adjacent cilia connected by a unique structure, the compartmenting lamella [22]. Compound cilia also occur in some single-celled ciliates like *Stentor* [23]. Table 1 summarizes the types of ciliation and ciliary movement across animals.

**Table 1.** Types of ciliation and ciliary movement (based on [15,16,24]).

animal group	Placozoa	sponges, cnidarians, flatworms, ectoprocts, cephalochordates	some bryozoan larvae	echinoderms, phoronids, brachiopods	molluscs, some bryozoans and rotifers, the annelid *Chaetopterus*	most spiralian phyla, some rotifers, tunicates	Ctenophores
ciliated surface	ciliated epithelia	ciliated epithelia	ciliated epithelia	ciliary bands	ciliary bands	ciliary bands	ciliated comb plates
ciliated cells	monociliated	monociliated	multiciliated	monociliated	multiciliated	multiciliated	multiciliated
type of cilia	separate	separate	separate	separate	separate	separate	compound
type of ciliary movement	uncoordinated	dexioplectic metachronal waves	dexioplectic metachronal waves	dexioplectic metachronal waves	laeoplectic metachronal waves	dexioplectic metachronal waves	antiplectic metachronal waves

### (b) Cilia in suspension feeding

Suspension feeding is widespread among larval and adult aquatic animals. Many animals have specialized ciliated structures like arms and tentacles to aid feeding, including the larvae of echinoderms, enteropneusts and lophophorates (brachiopods, phoronids, ectoprocts). Larval ascidians do not have ciliated feeding structures, but adults feed by filtering food particles through the branchial basket [25].

Feeding ciliary systems overlap with locomotory systems in some planktonic larvae with ciliary bands. There are two main suspension-feeding systems in these larvae: the upstream and downstream collecting systems. Larvae with one ciliary band use an upstream collecting system that concentrates food particles upstream of the ciliary band. Larvae with multiple ciliary bands rely on a downstream collecting system, also known as opposed-band feeding, where food particles are collected downstream of the main ciliary band [26]. Some planktotrophic pilidium larvae of nemerteans have ciliary bands, but they use muscular contractions of the lappet to induce local flexures of the ciliary band that efficiently funnel algae into the mouth [27].

### (c) Swimming–feeding trade-off

It has been suggested that larval forms, behaviours and preferred habitats result in part from a trade-off that exists between swimming and feeding. Feeding and swimming efficiencies depend largely on the length of cilia and the size of the ciliary bands [28]. Echinoderms, hemichordates and lophophorates have long ciliated arms or lobes and an upstream collecting system. In the case of these groups, the decreased feeding efficiency of short (20–25 µm) cilia on monociliated cells is compensated for by an extension in the size of the ciliary band. On the other hand, cilia on multiciliated cells are longer, have faster effective strokes and permit their carriers to feed using opposing flow currents between the opposing ciliary bands [28].

In the bipinnaria larva of *Patiria miniata*, a starfish that uses only one ciliary band for both swimming and feeding (upstream collecting system), it was demonstrated that the cilia can change stroke direction, generating different complex patterns of vortices depending on whether the larva swims or feeds [6].

## 3. Ciliary coordination by biophysical and cellular mechanisms

For directional movement, changes in motion and efficient filter feeding, the activity of beating cilia needs to be coordinated and regulated. Ciliary coordination can occur at different scales, from local coordination of adjacent cilia to the coordination of cilia on distant parts of the body (e.g. segmental ciliary bands). The coordination is owing to biophysical, cellular and neuronal mechanisms.

### (a) Metachronal waves

Most ciliated fields display metachronal waves, which are more efficient than non-metachronal beating in terms of energetics and flow generation [29,30]. Metachronal waves have an important contribution to swimming dynamics. The waves contribute to flow generation and could thus in principle exert a torque (turning force) on a swimming body. In addition, torque can also be generated by the azimuthal offset of the cilia [31]. The torque, together with the posterior-directed flow from effective ciliary strokes, generates the helical swimming trajectory characteristic of most larvae [32]. In helically swimming larvae, the direction of body rotation is usually opposite to the direction of wave propagation [24]. Understanding the generation of the different types of waves is an important future challenge for understanding ciliary coordination and swimming mechanics.

The direction of wave propagation relative to the effective ciliary stroke distinguishes four major forms of metachrony (figure 3). Symplectic waves propagate in the direction of the effective stroke and antiplectic waves in the opposite direction. Diaplectic waves are perpendicular to the effective stroke and can propagate either to the left (laeoplectic) or to the right (dexioplectic) [24]. In ciliary bands, the most common form of metachrony is dexioplectic, although some molluscs, bryozoans and larvae of the annelid *Chaetopterus* show laeoplectic waves [24]. Other exceptions include placozoans, where ciliary beating seems to be uncoordinated [15,16], and ctenophore comb cilia where the waves are antiplectic [15,16] (table 1).

Flow-based hydrodynamic coupling of adjacent cilia of the same ciliary band or the same ciliated epithelium contributes to the generation of metachronal waves. Mathematical models of ciliary beating and coordination are able to recapitulate metachronal synchronization [33,34]. In the unicellular green alga *Chlamydomonas reinhardtii*, basal-body coupling also contributes to ciliary coordination [35], but it is unclear whether this mechanism also occurs in ciliary bands in animals. In the comb plates of ctenophores, there is an additional level of short-range coordination, whereby adjacent cilia are directly coupled by filamentous bridges [17,22].

### (b) Gap junctions

In some ciliated surfaces, there are gap junctions facilitating electrical coupling between ciliated cells. This may allow the fast propagation of signals leading to the coordination of ciliary activity across cells [36]. In the tunicates *Oikopleura* [37] and *Corella* [38], water flow into the adult animal is aided by the beating cilia of the branchial sac. Some, but not all, of the ciliated cells are innervated, and gap junctions between the ciliated cells ensure rapid signal propagation and coordinated beating [38,39]. Gap junctions have also been identified via electron microscopy between velar ciliated cells in mollusc larvae [36] and comb plate ciliated cells of ctenophores [40].

## 4. Neuronal and paracrine mechanisms of ciliary coordination

Long-range ciliary coordination has been observed between different ciliary bands in many organisms. The coordination can extend to three different aspects of ciliary activity that cannot be fully accounted for by hydrodynamic coupling and gap junctions: simultaneous ciliary reversals, arrests and frequency changes [41]. In several instances, it has been noted that these events are influenced by neurotransmitters and neuropeptides and accompanied by calcium-dependent action potentials. Ciliary bands are innervated in many animals, and the activity of ciliomotor neurons, where demonstrated, controls the phenomena of long-range ciliary coordination. Below we discuss the types of phenomena where long-range ciliary coordination has been observed. We also discuss the neuronal or paracrine mechanisms that have been suggested to ensure coordination.

### (a) Coordination of ciliary closures

Coordinated closures have been observed in the ciliary bands of annelids [12,42], molluscs [36,43,44] and echinoderms [45], in the ciliated epithelia of placozoans [16], in the gill bar cilia of amphioxus [46] and in the branchial basket cilia of juvenile and adult tunicates [25,47]. The extent and duration of ciliary arrests can be varied and depend on the species and the developmental stage [48].

Alternating phases of spontaneous ciliary closures and beating control swimming depth in planktonic larvae [49]. Ciliary arrests also occur as part of startle and avoidance responses to mechanical stimuli [50,51] and in response to chemical stimuli, including settlement cues [43,52]. In hemichordates and echinoderms, mechanical stimulation leads to ciliary reversal or stoppage [45,51,53]. In the neuron-less placozoan *Trichoplax adhaerens,* the gliding movement halts when encountering food, likely owing to a pause in the activity of cilia [16].

The signalling mechanisms of ciliary closures have been studied in pharmacological, electrophysiological, calcium imaging and cell ablation experiments. Electrophysiological recordings revealed that ciliary closures are accompanied by bursts of membrane depolarization in the ciliated cells of larval annelids [8,49], molluscs [36,54], echinoderms [55] and the branchial baskets of adult tunicates [25]. The depolarizations lead to an increase in the concentration of intracellular calcium, as shown by calcium imaging in larval *Platynereis* [12].

Neurons that drive these ciliary depolarizations have been identified in larval *Platynereis* [12] and in the central ganglion of adult tunicates [25,38]. These two examples are also telling of the molecular mechanisms driving arrests.

Studies on *Platynereis* uncovered that the rate of change of intracellular calcium, and not absolute concentration, triggers closures. As long as the calcium concentration in the ciliated cells is increasing, the cilia remain arrested. Ciliary beating resumes when the calcium concentration starts decreasing [12]. The dependence of ciliary activity on the rate of calcium change was also shown to be important during sperm chemotaxis, suggesting a similar mechanism of adaptive signalling [56].

More information about the second messenger cascades involved in triggering ciliary closures came from pharmacological experiments in the tunicate *Ciona intestinalis.* In the *Ciona* branchial basket cilia, the calcium-dependent arrests are modulated by a pathway involving cAMP. It was shown that an increase in cAMP concentration reactivates the arrested cilia, which suggests there are antagonistic effects of calcium and cAMP [47].

While the details of signalling mechanisms driving ciliary closures remain largely unknown, some information is available about the neurotransmitters and neuromodulators that induce them. In larval *Platynereis* [8,12], cholinergic neurons were shown to induce closures. Pharmacological experiments in molluscs [44], the annelid *Spirobranchus* [42] and hemichordates [51] indicate that acetylcholine and probably also catecholamines may be responsible for inducing ciliary closure, while serotonin inhibits closures. In most of these experiments, it is difficult to distinguish direct neurotransmitter effects on the ciliated cells from potential indirect effects, for example, on presynaptic pacemaker systems.

Secreted peptides can also have an effect on ciliary closures. In *Trichoplax*, the coordinated ciliary pauses may be owing to diffusible neuropeptide-like molecules [57]. Treatment of *Platynereis* larvae with synthetic neuropeptides revealed that several peptides can induce or inhibit ciliary arrests [49,58]. The site of action of these neuropeptides is not known, but they may modulate the ciliomotor pacemaker circuit in these larvae [12]. Neuropeptides can modulate pacemaker systems as demonstrated, for example, in the crustacean somatogastric ganglia [59,60].

### (b) Coordination of ciliary reversals

Ciliary reversals, or reversals of the direction of the effective stroke of ciliary beating, have been observed in ctenophores and some deuterostomes (echinoderms and tunicates).

In ctenophores, ciliary reversals occur during prey capture [61]. Upon contact with prey, the ctenophore comb cilia briefly stop beating (quiescence). Quiescence is followed by a unilateral ciliary reversal in the ctene rows that were catching the prey. Reversals can also be induced by electrical, mechanical or chemical stimulation of some larval ctenophores [62]. Reversals were demonstrated to be calcium-dependent and triggered by voltage-dependent calcium channels [17,62].

In echinoderm larvae, contact with food particles leads to brief local ciliary reversals in the ciliary band [37,63]. Larger-scale, coordinated reversals are observed as an avoidance response upon contact with obstacles and they lead to the animal swimming backwards [45]. The reversals are accompanied by action potentials [55] and involve cholinergic and catecholaminergic neurotransmission [45,64,65]. Pharmacological experiments implicate an ionotropic (nicotinic) acetylcholine receptor in stimulating the avoidance response-related reversals [45]. However, specific ciliomotor neurons mediating this behaviour have not yet been identified.

In the branchial basket of the tunicate *Oikopleura*, coordinated reversals of ciliary beat in two ciliated rings induce a reversal of the water current through the pharynx [37]. The reversals are accompanied by membrane depolarizations of the ciliated cells. This happens spontaneously, as well as in response to mechanical or electrical stimulation. It is presumed that reversals increase in instances of greater particle density in natural conditions. The ciliated cells of *Oikopleura* are innervated with peripheral nerves. As spontaneous reversals continue after the removal of the brain, it was suggested that a peripheral pacemaker system exists to induce them [37].

### (c) Control of ciliary beat frequency

Similar to ciliary closures, ciliary beat frequency (CBF) can be modulated by neurotransmitters and neuropeptides to control swimming speed or feeding behaviour. Serotonin and dopamine are the two transmitters most commonly associated with a change in CBF. Serotonin generally increases CBF and inhibits closures. Dopamine most commonly decreases CBF, with a few exceptions.

Serotonin is the most common cilioexcitatory neurotransmitter in aquatic embryos and larvae. In encapsulated embryos of the gastropod *Helisoma*, specific serotonergic neurons mediate hypoxia-induced increases in CBF [66,67]. This induces rapid rotations of the embryos, and more efficient oxygen diffusion owing to increased stirring. This serotonin-mediated response acts through G-protein-coupled receptors. One receptor signals through the Gq pathway, leading to increases in intracellular Ca^2+^ [68]. The hypoxia response is also accompanied by increased cAMP levels in the ciliated cells, mediated by another, Gs-coupled serotonin receptor [69]. The different serotonin receptors may have a function during different phases of the behavioural response [69].

Similar cilioexcitatory effects of both serotonin and cAMP were demonstrated in pharmacological experiments in annelids [12,49] and echinoderms [70,71]. Serotonin treatments also lead to increased CBF in mollusc velligers [44] and echinoderm plutei [72]. In a rare example of surface ciliation in a vertebrate, the CBF of *Xenopus laevis* epidermal larval cilia is controlled by serotonin secreted from specialized epidermal cells binding to the ionotropic 5-HT3 receptor on ciliated cells [73]. Serotonin was found to have cilioexcitatory effects in other vertebrate tissues as well, including the mouse trachea [74] and rat ependymal cells, where the cilioexcitatory effects are calcium-dependent [75].

Dopamine was demonstrated to decrease CBF in pharmacological experiments on echinoderm plutei and bipinnariae [53,70], mollusc veligers [44] and annelid trochophores [42]. In all these species, dopamine treatment also induces more frequent ciliary closures. As an exception, in the embryos of the snail *Lymnaea*, dopaminergic neurons seem to induce CBF increases during the hypoxia response [67]. In sea urchin embryos, dopamine increases swimming speed likely through a cilioexcitatory effect [70,76]. Experiments in echinoderms suggest a role for acetylcholine, adrenaline and noradrenaline in decreasing CBF [53,70].

In addition to neurotransmitters, neuropeptides also exhibit stimulatory and inhibitory effects on CBF. In *Platynereis,* 9 of 11 neuropeptides tested were found to have a cilioexcitatory effect, while the remaining two neuropeptides reduced CBF [49]. Neuropeptide antibody stainings have revealed peptidergic nerves along ciliary bands in several larvae. RFamide-like neuropeptides are commonly detected along ciliary bands [44,67,77–80]. In *Platynereis* larvae, FMRFamide increases CBF and leads to higher positioning in the water column*,* while in the *Crepidula fornicata* veliger, it has the opposite effect [44]*.* In the nemertean *Lineus longissimus,* two neuropeptides (excitatory peptides 1 and 2) increase CBF [81]. While the influence of peptides on CBF has not been explored in vertebrates in great detail, it has been shown thus far that the melanin-concentrating hormone exhibits cilioexcitatory effects in the mouse ependymal cells [82].

The signalling cascades involved in coordinated changes in ciliary activity generally involve calcium as a second messenger. The diverse effects of calcium on ciliary activity may partly be owing to differences in calcium channels, signal location or dynamics, or interactions with other second messengers. For example, the fine-tuning of ciliary closure dynamics is achieved through an antagonism between calcium and cAMP signalling in *Ciona* [47]. CBF is generally regulated by cAMP (e.g. [49,71]) and may interact with calcium to fine-tune responses. In addition, the rate of change in calcium concentration can also be important [12,56]. Finally, different processes rely on different calcium channels. Ciliary reversals are mediated by voltage-dependent calcium channels [62]; CBF changes can be triggered through Gq signalling and the inositol trisphosphate receptor [68].

## 5. Innervation of ciliary bands

The phenomena of long-range ciliary coordination discussed above are commonly under neuronal control.

The most unambiguous data about the innervation of larval ciliary bands are available from electron microscopy studies. Electron microscopy enables the identification of neurons forming synapses on ciliated cells. Synapses from nerves running along ciliary bands or ciliated epithelia have been described in ctenophores [62], the larvae of platyhelminths [83], annelids [12,48,84], molluscs [54], nemerteans [85] and echinoderms [53].

The axons of neurons that synapse on ciliated cells run along the ciliary bands. In some cases, these nerves form a distinct ciliomotor nervous system that is clearly distinguishable from the central nervous system. The best example of a distinct ciliomotor nervous system can be found in the Mueller's larva of the polyclad flatworm *Pseudoceros canadensis*, which has a unique intraepithelial nervous system associated with the ciliary band [83]. The ciliomotor nervous system is separated from the central nervous system by the basement membrane and there are only two points of contact between the two systems. Many of the cells of the ciliomotor nervous system are bipolar sensory cells with sensory dendrites among the cells of the ciliary band. Pilidium larvae of nemerteans also have a distinct ciliomotor nervous system. In these larvae, the main ciliary band is innervated by the marginal nerve, the largest nerve in the body. Additional nerves connect the marginal nerve to the oral nerve that innervates the accessory oral ciliary bands [85,86].

Further knowledge about the innervation of ciliary bands comes from immunofluorescence or histological stainings. Serotonin immunoreactivity has been detected in the ciliary nerves in most groups of ciliated animals (table 2; [101]). Glyoxal-induced fluorescence imaging also shows catecholamine presence in the ciliary band nerves of nemerteans [86], annelids [108], phoronids [109], echinoderms [110,111] and enteropneusts [105]. Cholinergic innervation has been characterized in ciliary bands of echinoderms [112], enteropneusts [105], annelids [12] and molluscs [113].

**Table 2.** Summary of ciliation and the neuronal control of cilia across metazoans. DA, dopamine; NPs, neuropeptides; PKC, protein kinase C.

organism	species	developmental stage	ciliated cells	ciliary bands	innervation	signalling	neurotransmitters	neuropeptides	CBF (Hz)	arrests	sensory input
sponges	*Amphimedon queenslandica* [87,88]	parenchymella larva	all larvae have monociliated epithelial cells, except hexactinellid trichimella larvae (multiciliated) [89]	rows of cilia on larval surface except for pole				no neuropeptides found in the genome			negative phototaxis
placozoans	*Trichoplax adhaerens* [16,57]	adult	monociliated	ciliated epithelium	none		none	FFNPa, ELPE, MFPF and WPPF cause cilia to pause and the animal to flatten, diverse effects		when feeding	food
ctenophores	various [17,62,90,91]	adult, cydippid larva	multiciliated, filamentous bridges between cilia facilitate mechanical coordination	8 paired ciliated comb rows	beating usually initiated at the pacemaker balancer cilia in the aboral statocyst; synapses shown onto ciliated cells	elevated Mg levels abolish ciliary function, implying Ca-signalling	only Glu, no other classic neurotransmitters	several ctenophore-specific neuropeptides	7 (*Martensia ovumi),* 14–15 (*Beroe),* 5–13 (*Leucothea pulchra*)	arrests upon stimulation; quiescence and ciliary reversals during prey capture	mechanical, chemical or electrical stimuli inhibit ciliary movement
cnidarians	*Tripedalia cystophora* [92]	planula	monociliated	ciliated epithelium	not known						ocelli (directional light signals) used for steering swimming
molluscs	*Calliostoma ligatum* [36]; *Phestilla sibogae* [43,52]; *Haliotis rufescens* [93]; *Crepidula fornicata* [44]	veliger	multiciliated	velar cilia		Ca-dependent action potentials lead to arrest; settlement-induced arrests mediated through GABA; gap junctions between cilia	5HT increases CBF, abolishes arrests; DA increases CBF	FMRFamide decreases CBF, larvae lower in water column	5–7 Hz	spontaneous and induced	responds to dissolved settlement cues (prey extract) with arrests
	*Helisoma trivolvis* [66,94,95]	early embryo (no larval stage)	multiciliated	pedal and dorsolateral (prototrochal) ciliary bands	serotonergic sensory-motor ENC1 neurons; type 5 and 7 receptors in the foot ciliated cells	Ca-signalling through PKC	serotonin increases CBF				ENC1 sensory-motor neurons directly respond to hypoxia —acceleration in rotational swimming
	*Lymnea stagnalis* [67]	early embryo (no larval stage)	multiciliated	ciliated apical plate region, pedal and dorsolateral (prototrochal) ciliary bands	transient apical catecholaminergic (TAC) neurons	dopamine may act on D1 receptor	serotonin and dopamine increase CBF	FMRFamide in TAC neurons	∼14 Hz in pedal cilia		dopaminergic and serotonergic neurons respond to hypoxia —acceleration in rotational swimming
annelids	*P. dumerilii* [12,49,50]	trochophore	multiciliated	prototroch and metatroch	full ciliomotor circuit reconstructed	Ca-dependent action potentials	5HT increases CBF, catecholamines decrease it	RYa, FVMa, DLa, FMRFa, FVa, LYa, YFa, L11, and SPY increase CBF, FLa and WLD decrease CBF; RYa, FVMa, DLa, FMRFa and FVa reduce arrests, FLa, WLD and MIP increase arrest	∼15 Hz	spontaneous and induced; 5HT decreases closure frequency	phototaxis, startle response, settlement-induced arrests
	*Capitella teleta* [96] *Spirobranchus giganteus* [42]; *Phyllodoce* sp. [48]	trochophore	multiciliated	prototroch and metatroch	prototroch nerve	Ca-dependent	β-blockers (alprenolol) lead to arrest	DLamide, FVamide, RYamide immunoreactivity in apical organ neurons with projections to ciliary band		yes, partial in *Phyllodoce*	
	*Owenia fusiformis, Owenia collaris* [15]	mitraria larva	monociliated tentacle cells	primary ciliary band with 2 rows of cells, later also secondary ciliary band on posterior end							
nemerteans	*Lineus albocinctus, Micrura purpurea* [86]; *L. longissimus* [81]; *Micrura alaskensis* [27,97]	pilidium	multiciliated		marginal nerve (5HT); peptidergic (EP) nerves projecting from apical organ to the nerves underneath ciliary bands			2 excitatory NPs (EP1, EP2) increase CBF	9.6 Hz (apical) and 10.3 Hz (lateral ciliary band)	in response to feeding	arrests upon mechanosensory stimuli related to feeding
platyhelminths	*P. canadensis* [83]; *M. crozieri* [79]	Mueller's larva	multiciliated	ciliary band	ciliary nerve		5HT immunoreactivity in ciliary band nerve	FMRFa immunoreactivity in ciliary band nerve		no	
cycliophorans	*Symbion pandora* [98,99]	chordoid larva	multiciliated	2 ventral anterior bands, ciliated body field, ciliated foot			no 5HT immunoreactivity in anterior ciliary bands				
bryozoans (ectoprocts)	*Fredericella sultana* (phylactolaemate) [77]	larva	multiciliated	ciliated epidermis			5HT and DA stimulate negative phototaxis	FMRFamide immunoreactivity in ciliated cells			phototaxis
	*Flustrellidra hispida, Bugula fulva, Alcyonidium gelatinosum, and Bowerbankia gracilis* (gymnolaemate) [78]; *Cryptosula* sp. [96]; *Bugula neritina* [100]	coronate larva	multiciliated	1 ciliary band (corona)				FMRFamide immunoreactivity in ciliated cells, RYamide immunoreactivity in lateral cells projecting to ciliary band			
entoprocts	various [101,102]	swimming-type larva	multiciliated	prototroch, metatroch, ciliated food groove and gastrotroch	prototroch nerve		no 5HT immunoreactivity along ciliary band				
phoronids	*Phoronis muelleri* [15,80]	actinotroch	multiciliated	preoral, postoral and tentacle ciliary bands; archaeotroch on posterior end; all monociliated			5HT-like immunoreactivity in tentacles and archaeotroch	FMRFamide immunoreactivity in ciliated cells near neurophil			
rotifers	various [15,103]	adult	multiciliated	3 ciliary bands: trochus, circumapical field and cingulum; pseudotrochus in other species	no known innervation						
brachiopods	*T. transversa* [104]	larva	monociliated	ciliary bands				FMRFamide induces defence behaviour (sinking)			defence response to mechanical stimuli
echinoderms	*Psammechinus miliaris* [70,71]; *Pseudocentrotus depressus, Hemicentrorus pulcherrimus* [70,72]; *Lytechinus pictus* [45]	pluteus	monociliated	1, circumoral		excitatory role of cAMP, Ca involved in both excitation and inhibition; suggested that nicotinic AChR is involved	5HT and beta-adrenergic agonists increase, DA decreases CBF, DA, adrenaline and cholinergic agents cause ciliary reversal and arrest			coordinated arrests and reversals	avoidance response
	*Pisaster ochraceus* [53]	bipinnaria	monociliated	1, circumoral	ciliary nerve; aminergic sensory cells		cholinergic agents, DA and adrenaline reduce beating			no, no reversals either	avoidance response (reduced ciliary beating)
hemichordates	*Balanoglossus biminiensis* [51]; *Balanoglossus proterogonius* [105]	tornaria	monociliated cells in the two circumoral bands, multiciliated in teletroch	two circumoral bands, teletroch	innervated in part by fibres from the apical plate and adoral nerve centres; unknown teletroch innervation		cholinesterase activity in the epithelium along the length of the oral ciliary bands, but not in the telotroch; single catecholaminergic cells in postoral band and teletroch; cholinergic agents induce teletroch arrest			Yes (15–20 s). Some parts of the telotroch may stop beating while others continue	avoidance response
tunicates	*Ciona intestinalis* [47], *Chelyosoma productum* [25]	adult	multiciliated	around the stigmata of the branchial basket (stigmatal ciliary system)	ciliary arrest (CA) neurons (part of the visceral nerve of the central ganglia) directly controlling ciliary arrests	Ca-dependent action potentials lead to arrest; cAMP activates quiescent cilia				spontaneous or in response to mechanical, electrical, or chemical stim- ulation; 1–2 s in duration	
cephalochordates	*Branchiostoma floridae*	larva [106]	monociliated	ciliated epidermis; loss of cilia from 24 h post-fertilization							
		juvenile [46,107]	monociliated	gill bar lateral cilia	neuronal control confirmed; innervation by atrial nervous system			FMRFamide immunoreactivity in the atrial nervous system innervating the cilia			

It was shown through these tissue stainings that the ciliated velum of the mollusc veliger is innervated by bipolar and tripolar cholinergic neurons. Bipolar neurons were found at the base of the velum, connecting it with the cerebral ganglia [113].

## 6. The ciliomotor circuit in the *Platynereis dumerilii* larva

**Figure 4.**
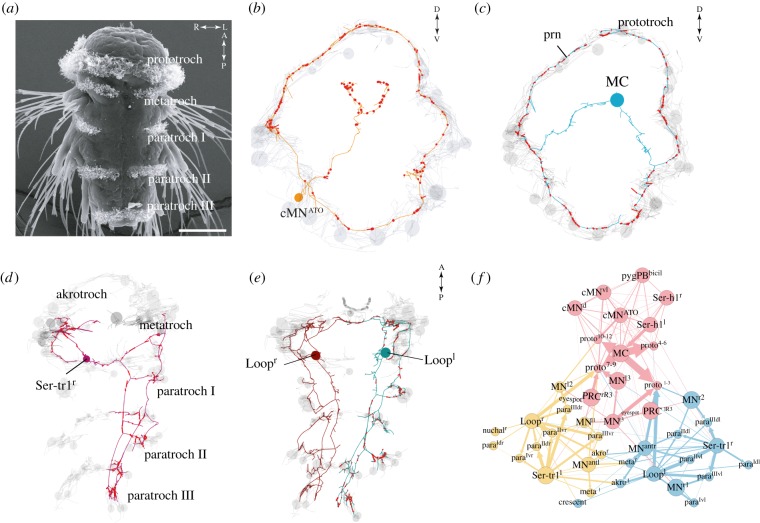
The ciliomotor circuit of the *Platynereis* larva. (*a*) SEM of a *Platynereis* nectochaete (3 days old) larva with ciliary bands labelled. Scale bar 50 µm. (*b*) serial scanning transmission electron microscopy (ssTEM)-based reconstructions of one of three catecholaminergic neurons (anterior view) and (*c*) of the closure-inducing cholinergic MC neuron (anterior view) in the *Platynereis* ciliomotor circuit. Ciliated cells are shown in grey. (*d*) Reconstruction of the serotonergic Ser-tr1 and (*e*) cholinergic Loop ciliomotor neurons (ventral views). (*f*) Synaptic connectivity graph of all ciliomotor neurons and ciliary band cells.

The most comprehensive characterization of ciliary band innervation comes from the reconstruction of the ciliomotor nervous system in the *Platynereis* nectochaete larva [12]. Here, all neurons that synapse on locomotor cilia have been reconstructed by serial electron microscopy (figure 4). The neurons form a distinct ciliomotor circuit with a function in the control of ciliary closures and beating. Most ciliomotor neurons are morphologically unique and have two axons emanating from the cell body. These neurons are the largest in the body, with very long axons, spanning the entire prototroch ciliary band or all segmental ciliary bands [12]. Through immunofluorescence, *in situ* hybridization and transgenesis, the *Platynereis* ciliary neurons have been classified into 11 cholinergic, five serotonergic and three mixed peptidergic–catecholaminergic neurons [12].

The 3-day-old *Platynereis* larva (nectochaete) has multiple segmentally arranged ciliary bands, where the beating and closures show a rhythmic pattern and cross-band synchronization. Imaging of neuronal activity reported by the calcium sensor GCaMP6 showed that the activity of the serotonergic ciliomotor neurons correlates with ciliary beating, whereas cholinergic neurons are active during closures. Laser ablation of a major head cholinergic neuron (MC neuron, figure 4) abolished the rhythmic closures of the main ciliary band innervated by this neuron.

The ciliomotor circuit is under the control of a central pattern generator (CPG), the ciliomotor pacemaker. The three peptidergic–catecholaminergic neurons of the ciliomotor circuit activate rhythmically and likely form the pacemaker. Two of them are active during ciliary closures and one during the phases of beating. This rhythmically active circuit driving alternating phases of swimming and sinking (during closures) may enable the larvae to maintain a constant depth in the water column [49].

The activity of this pacemaker seems to be under the influence of different neuropeptides and hormones released in response to sensory cues or following a circadian rhythm. Several neuropeptides expressed in sensory–neurosecretory neurons in the larval brain influence larval vertical distribution through changing the ciliomotor rhythm (inhibiting or stimulating ciliary closures) [96]. A reduction in closures moves the larvae upwards in the plankton, whereas more frequent closures lead to sinking. Sensory cues may trigger neuropeptide release and concomitant changes in ciliary closures. For example, during larval settlement, chemical cues likely lead to a release of myoinhibitory peptides from chemosensory–neurosecretory neurons [114]. Exposing larvae to these peptides increases ciliary closures, which causes the larva to sink.

The frequency and duration of ciliary closures also change in a diurnal cycle, with more frequent closures occurring during nighttime. This effect may be mediated by melatonin signalling acting on cholinergic ciliomotor neurons [8].

*Platynereis* larvae also respond to vibrational stimuli by ciliary arrests [50]. The stimuli are detected by ciliated mechanosensory neurons called the collar-receptor neurons (CRs). CRs synapse on different interneurons that in turn synapse on the cholinergic intersegmental ciliomotor neurons. This feed-forward circuit can explain how a vibrational stimulus leads to the coordinated arrest of all locomotor cilia in the larva.

## 7. The evolution of ciliomotor cell types and circuits

**Figure 5.**
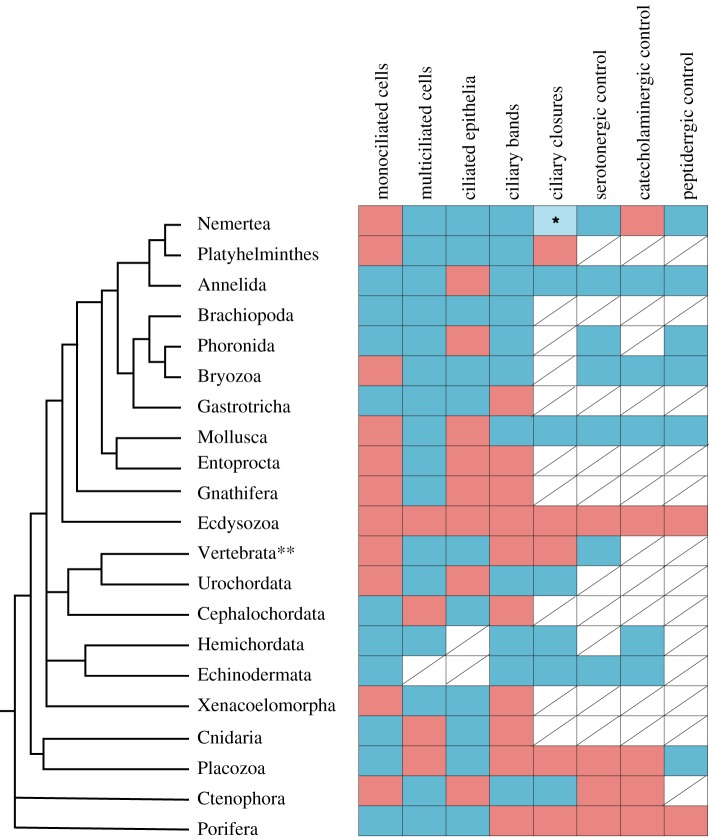
Types of invertebrate ciliary systems and their control. Blue squares indicate presence, and red squares absence of a trait. Squares with no available data are crossed out. Phylogeny is based on [115–117]. *Nemerteans show brief arrests coupled with muscle contractions upon contact with food particles. **Only motile cilia on the body surface (anuran larvae) are considered.

We can note several general principles and similarities in the regulation of ciliary locomotion across different groups of animals (figure 5). To achieve coordinated movement of cilia across longer distances, neuronal input is required and achieved through the release of neurotransmitters and neuropeptides. Even in placozoans—animals that lack a nervous system—a function for neuropeptides in stopping ciliary gliding has been confirmed. In different groups where their effects were studied, ciliary responses to neurotransmitters were shown to be similar. Serotonin application increases CBF and decreases the occurrence of ciliary closures, while by contrast, acetylcholine and catecholamines decrease CBF and increase closures [12,42,44,66,70,72].

The general involvement of serotoninergic and catecholaminergic neurons in ciliary control suggests that such ciliomotor neurons may trace back to the protostome–deuterostome common ancestor.

In animals where neuronal control of ciliary activity has been demonstrated, such as molluscs, annelids, nemerteans, echinoderms and chordates [12,25,36,42–45,47], spontaneous ciliary closures have been recorded, implying the existence of a pacemaker system (CPGs) involved in generating the rhythm of beating versus closure, similar to *Platynereis* larvae [12]. Changes in the pattern of closures were shown to be induced by chemical [8,52] and mechanical [45,51,53] stimuli, suggesting that there is sensory innervation modulating the presumptive pacemaker function.

Apart from the pacemaker neurons in *Platynereis*, we know very little about the generation of ciliary rhythms in other animals. Electron microscopic studies identified large and morphologically distinct neurons in other larvae that span the whole body to innervate ciliated cells [83,85,86].

We hypothesize that ciliomotor neurons are special and form a distinct part of the nervous system with a unique function and evolutionary history. We call this the ciliomotor nervous system. In the *Platynereis* larva, the comprehensive characterization of the ciliomotor nervous system revealed many unique characteristics. First, all ciliomotor neurons, with two exceptions, have a unique biaxonal morphology where two axons emanate directly from the neuronal soma and project in two directions. Second, the ciliomotor neurons show a distinct activity profile that drives ciliary activity. Third, the ciliomotor nervous system has a unique connectivity pattern and forms a distinct subnetwork in the larval nervous system. Fourth, the ciliomotor system must be specific to the larval stages as ciliation and ciliary swimming are lost in the juvenile worms and are absent from adults. The developmental fate of the ciliomotor neurons is not known, but they will either disappear or completely change function.

In agreement with what we have found in *Platynereis*, a morphological reconstruction of the ciliomotor system in the platyhelminth Muller's larva by Lacalli [83] revealed that the ciliomotor system in this larva is clearly distinguishable from the central nervous system. In the pilidium larva, the largest and most distinct neuron innervates the ciliary margin. Giant serotonergic neurons with bi- or multiaxonal morphology have also been described in the phoronid larva [118]. These studies suggest that ciliomotor nervous systems form a distinct part of larval nervous systems, with unique characteristics and potentially a unique evolutionary history.

From the perspective of comparative neurobiology, ciliomotor neural circuits represent an interesting model system as they can be unambiguously identified through cell tracing in electron microscopy datasets (tracing backwards from ciliated cells). Such connectomic reconstructions of the circuitry underlying ciliary coordination in different animals would be valuable to understanding the evolution of these systems. Unravelling the evolution of ciliomotor circuits will also require research into the function and molecular specification of the cell types composing these circuits. This would require a combination of behavioural experiments, functional imaging (e.g. using genetically encoded calcium indicators) and genetic approaches. For example, an exciting subject of cell-type and circuit evolution would be a comparison of the annelid larval circuit to circuits in mollusc ciliated larvae. Both larval types show spontaneous and mechanically induced coordinated synchronized arrests that extend to all cilia, which suggests that a similar pacemaker operates in these larvae. In addition, the main ciliary band (prototroch) of annelid and mollusc larvae are likely homologous as they derive from the same blastomeres during the spiral cleavage pattern [119–121]. More generally, it would be interesting to study how ciliomotor systems compare across lophotrochozoan larvae. What are the differences between larvae with distinct ciliary bands and uniformly ciliated larvae? How is the nervous system in larvae with ciliary bands made of multiciliated or monociliated cells? How do systems regulating locomotory ciliary bands and feeding ciliary bands compare to each other?

Ciliomotor cell types likely coevolved with ciliary bands and their comparative study across animal groups may also reveal which larval types are homologous and which evolved independently. These are exciting questions for future neuro-evo-devo studies.

## Data accessibility

This article has no additional data.
